# Autism and Overcoming Job Barriers: Comparing Job-Related Barriers and Possible Solutions in and outside of Autism-Specific Employment

**DOI:** 10.1371/journal.pone.0147040

**Published:** 2016-01-14

**Authors:** Timo Lorenz, Cora Frischling, Raphael Cuadros, Kathrin Heinitz

**Affiliations:** Department of Education and Psychology, Freie Universität Berlin, Berlin, Germany; Harvard Medical School, UNITED STATES

## Abstract

The aim of this study was to discover how individuals with autism succeed in entering the job market. We therefore sought to identify expected and occurred barriers, keeping them from taking up and staying in employment as well as to identify the solutions used to overcome these barriers. Sixty-six employed individuals with autism–17 of them with autism-specific employment–participated in an online survey. Results showed a variety of possible barriers. Individuals in autism-specific employment named formality problems–problems with organizational and practical process-related aspects of the job entry–most frequently while individuals in non-autism-specific employment mentioned social problems–obstacles concerning communication and human interaction–most. In terms of solutions, both groups used their own resources as much as external help, but differed in their specific strategies. In addition, correlations of an autism-specific employment with general and occupational self-efficacy as well as life and job satisfaction were examined. Possible implications of the results are discussed with regard to problem solving behavior and the use of strengths.

## Introduction

Autism, from its first mention in the 1940s [1, 2], has since become a condition arousing interest not only in researchers and the public media but in employers as well. Companies in the IT sector such as “specialisterne” in Denmark, “Passwerk” in Belgium, or “auticon” in Germany specifically employ individuals with autism. However, as the Secretary General of the United Nations Ban Ki-moon pointed out recently, the overall majority of individuals with autism is still unemployed [3].

### Autism

According to the fifth edition of the Diagnostic and Statistical Manual of Mental Disorders (DSM-V), individuals with Autism Spectrum Disorder (autism) show repetitive behavioral patterns and impairment in communication skills from early childhood on [4]. The distinction between Autism Spectrum Disorder, Asperger’s Syndrome and Pervasive Developmental Disorder not otherwise specified in the previous editions has thus been replaced by an umbrella category that includes all three forms while differentiating by severity within the category. For the sake of non-discriminating language, we will use the term “Autism Spectrum Condition” and refer to people concerned as individuals with autism throughout the remainder of this manuscript.

Prevalence for Autism Spectrum Condition varies, but it is currently best estimated at 74 out of 10,000 children [5]. There is a positive correlation with prevalence and publication year of the study, which might stem from changes in the availability of diagnosis and services or changes of criteria for diagnosis rather than from an actual increase in cases of Autism Spectrum Condition [5]. Regardless, the importance of its related issues, like employment, rises with the prevalence.

### Autism Spectrum Condition and Employment

There is no all-embracing statistic as to how many individuals with autism are currently employed. So far, studies have usually assessed employment only in specific groups [6, 7], not permitting a broad generalization. An employment rate of one third has been found for young adults in the United States [8] and for adults in the United Kingdom [9]. The United Nations recently specified an employment rate of 20% [3].

However, research on reasons for the overall low employment rates or success stories of individuals with autism has been limited until now. To our knowledge, no study has been conducted that takes into consideration the ways in which the individuals overcame the barriers they encountered.

A possible explanation for the low employment rate could be barriers during job search, job application, or employment. Based on the outcome of their interviews, Müller, Schuler [10] identified such barriers in the categories of the application process (résumés, phone contact, interviews), the adaptation to new job routines, communication, and social interaction.

Several forms of employment for individuals with autism have been established. Frequently studied on different levels are competitive employment (regular job without support, non-autism-specific employment), supported employment (competitive employment with support by the employer or an agency, autism-specific employment) and sheltered workshops (long-term placement for individuals with disabilities; see [11] for an overview). In order to gain knowledge about possible barriers that limit access to the job market, we sought to qualitatively compare the reports given by individuals in autism-specific employment with individuals with autism working in non-autism-specific employment.

### Positive Psychology and Focus on Solutions

Focusing on strengths instead of weaknesses is the central idea of the concept of positive psychology [12] and the strengths approach [13]. Applied to organizational psychology, this suggests the importance of identifying and fostering the positive capabilities of individuals rather than trying to erase weaknesses [14]. Based on these reflections of positive organizational psychology, we laid our focus on successfully employed individuals with autism in order to identify not only barriers, but also solutions used to overcome them. This might lead to the identification of important strengths that can be used for practical application, i.e. to create a basis for potential interventions both at the workplace and in support programs. With the help of these findings and the results of the positive constructs we measured, we hope to encourage individuals with autism, employers, and support workers to focus on strengths when paving the way for employing individuals with autism.

### Self-efficacy

In research on people’s behavior towards overcoming job barriers (e.g.[15]) as well as raising their life satisfaction [16], one concept has been focused on: self-efficacy–one of the four components of psychological capital [17]. It is a construct of social cognitive theory defined as “people’s judgments of their capabilities to organize and execute courses of action required to attain designated types of performances” [18]. Thus it is action-related and focuses on the future.

In neurotypical adults–those who show no divergence in neurological development viz. without autism [19]–positive correlations have been found between self-efficacy and work-related outcomes such as job performance, job satisfaction [20], health outcomes [21] and subjective well-being [22].

We assume that an autism-specific employment creates a more supportive environment than a non-autism-specific employment. As a result, this support may lead to higher self-efficacy because such employees are supported to experience mastery and receive verbal persuasion, both of which are important sources of self-efficacy [23].

### Life and Job Satisfaction

Life satisfaction is a concept closely associated with subjective well-being [24]. As opposed to the emotional components of subjective well-being, life satisfaction “should be viewed as a global assessment of a person's quality of life according to his own chosen criteria” ([25]; p. 478]).

Overall job satisfaction, as a measure of work-related subjective well-being, evaluates one’s job affectively [26]. It has been closely related to one’s overall satisfaction with life and job performance [27]. To our knowledge no study on the relation between employment and life satisfaction or job satisfaction in individuals with autism has been conducted up to this point.

We assume that an autism-specific employment creates a better person-organization and person-environment fit than a non-autism-specific employment. As a result, this fit may lead to a higher life and job satisfaction [28–31]. Supported employments have previously been found beneficial for individuals with autism, relating to improvement in cognitive skills even outside the work domain [32] and in quality of life [33, 34].

## Methods

### Participants

Participants in this study were recruited through autism community forums and through internal communication of the survey in an autism-specific company. Selection criteria for this study were as follows: (1) a formal diagnosis of autism and a score of ≥6 on the Autism Spectrum Quotient Test with 10 items (AQ-10) [35] and (2) current employment. 16 participants had to be excluded because they did not meet these selection criteria (14 due to a missing diagnosis, one due to a score of <6 on the AQ-10 and one due to being unemployed). The AQ-10 was conducted in order to affirm the self-reported diagnosis of autism. Since formal diagnosis of autism was a necessary criterion for employment at the autism-specific company, no AQ-10 was tested in this group.

Participants in this study were 66 German individuals with autism (females: 36; males: 29; other: 1).

The participants’ age ranged from 22 to 55 (M_age_ = 35.96; SD_age_ = 10.22). All participants were employed and their mean for organizational tenure was 4.68 years (SD = 6.55 years). Forty-nine of them were in non-autism-specific employment (females: 36; males: 12; other: 1) and 17 in autism-specific employment (all male). An overview of the occupational fields of all participants can be found in Table 1. All participants in the autism-specific company were employed in a company in the field of IT, thus they were sorted into the category of natural sciences, geography and computer science.

**Table 1:** Current employment of individuals with autism.

Classification according to KldB 2010 code	N	%
Agriculture, forestry, animal husbandry, and horticulture	1	1.5
Production of raw materials	3	4.5
Construction, architecture, surveying, and building technology	2	3
Natural sciences, geography and computer science	25	38
Transportation, logistics, protection and security	2	3
Commercial services, retail, sales and distribution, hotels and tourism	7	10.5
Business organization, accounting, law and administration	9	13.5
Health care, social affairs, and education	12	18
Humanities, social sciences, and economic sciences, media, art, culture and design	5	8

*Note*. KldB = Klassifikation der Berufe (classification of occupations)

The survey was administered in German. Participation in this study was completely voluntary including informed consent. All individuals participated via an online survey they could take at a time of their liking. They were informed that their data was obtained and analyzed anonymously and that they could interrupt or stop the survey at any time.

### Qualitative measures

#### Materials

We created a qualitative questionnaire with a total of 28 open-formatted questions. We constructed these questions forming eight thematic blocks about topics that might influence the employment process of individuals with autism. Some of these topics had been introduced previously in interviews with autistic students and adults with work experience [10, 36]. Our thematic blocks addressed the topics of the general process of job-seeking, drafting applications, contact with employers, job demands, the workday, workplace equipment, work environment, support mechanisms, and other problems than those mentioned.

In each thematic block we asked individuals (1) what problems they expected regarding the particular topic, (2) which problems actually occurred and (3), if applicable, how they had solved these problems. Distinguishing the most important problems from the most frequent ones is fundamental for possible practical implications. Therefore, we asked participants to identify the three problems, which seemed most important to them, naming the most crucial first. An English version of the questionnaire can be found in S1 Appendix.

#### Data analysis

Responses were analyzed using inductive category formation in QCAmap by Mayring [37]. All responses were reviewed and main categories and subcategories were formed. These categories were the same for the expected and occurred problems. Different main and subordinate categories for the solutions were created. In a next step, all responses were coded independently by three raters, with the instruction to note problems with the coding for a review of the category system. Ambiguous categories were subsequently defined more explicitly and new categories were formed in order to relieve the “other” category. After a second coding, the system was reviewed again for possibly scarce definitions. One category was eliminated because it was used disproportionately little by all raters and there was consent as to how these responses could be coded instead. We measured the agreement between the three raters using the Fleiss’ Kappa coefficient for three or more raters as proposed by von Eye [38]. Our agreement was κ = .96 for expected barriers, κ = .93 for occurred barriers, and κ = .89 for solutions, resulting in a high mean agreement of κ_mean_ = .92 (*SD* = 0.03). All coding results depict the coding decisions of the main rater.

Answers were rated as irrelevant / not codable (1) when they were not comprehensible, e.g. contained only special characters like a question mark or an incomplete word sequence and (2) when they did not contain a response to the question, e.g. did not contain a barrier or a solution, respectively. The irrelevant answers in expected barriers (4%), occurred barriers (2%) and solutions (17%) were excluded from further analysis.

### Quantitative measures

#### Demographics

We collected data regarding age, gender (“male”, “female”, and “other” in order to include individuals that did not see themselves in one of the dichotomous categories), current employment, and the tenure in the current job. The current employment was encoded into fields of occupation in accordance with the”Classification of occupations” (Klassifikation der Berufe, [39]).

#### General self-efficacy

General self-efficacy was assessed using the General Self-Efficacy Scale developed by Schwarzer and Jerusalem [40]. The scale consists of 10 items (e.g. "I have no difficulties realizing my intentions and goals.") with a four-point Likert-scale ranging from 1 = “*I completely disagree”* to 4 = “*I completely agree”*. Cronbach’s α was .88.

#### Occupational self-efficacy

Occupational self-efficacy was measured using the Occupational Self-efficacy Scale [41]. The scale consists of 8 items (e.g. “I have a solution for every problem at my job.”) with a four-point Likert-scale ranging from 1 = “*I completely disagree”* to 4 = “*I completely agree”*. Cronbach’s α was .89.

#### Life satisfaction

Life satisfaction was measured with a German translation of the Satisfaction with Life Scale [42]. The scale consists of 5 items (e.g. “I am satisfied with my life.”) with a five-point Likert-scale ranging from 1 = “*I completely disagree”* to 4 = “*I completely agree”*. Cronbach’s α was .91.

#### Job satisfaction

Job satisfaction was measured with a German translation of three items proposed by Judge, Boudreau [27]. The items were as follows: (1) a yes-no response to the question “All things considered are you satisfied with your job?”, (2) a five-stage rating question “How satisfied are you with your job in general?” ranging from 1 = “*very unsatisfied”* to 5 = “*very satisfied”*, and (3) an item were the participants reported the percentage of time they were satisfied, dissatisfied, or neutral regarding their job. Due to their different response formats the items were standardized before further analysis. Cronbach’s α was .85.

#### Control Items

In addition to the questionnaires, participants completed the following control items:

1. My quality of life has improved since entering my current employment. (improvement item)
2. A job is important for my quality of life. (job importance item)
3. I can use and hone my strengths in my current employment. (strengths item)

Participants assessed their agreement with these statements on a five-point Likert-scale ranging from 1 = “*I completely disagree”* to 5 = “*I completely agree”*. These items were used in order to further determine possible underlying mechanisms of our expected trends and thus indicate a way for possible future research.

#### Data Analysis

Due to the small sample size we decided to use Bayesian data analysis instead of traditional frequentist analysis for more reliable results [43–45] and non-normal variables [46]. In Bayesian analyses, the data is combined with reasonable prior knowledge about the parameter in question which results in robust estimations, even in small samples when traditional approaches yield large standard errors and thus statistically less stable results. We ran our data analysis using R [47] with the R-package “Bayesian First Aid”[48] and WinBUGS [49]. The package uses an non-informative prior with a very broad t-distribution [45]. This use of an uninformative prior allows the estimation to closely mimic classical frequentist estimation as the prior does not influence the results while resulting in more intuitive and robust inferences on parameters [50]. Group differences in answer frequencies of open questions were evaluated by Bayesian inference tests using binomial distribution. The posterior distribution was inspected to find the percentage in favor of a difference hypothesis. In Bayesian correlations we report mean scores of parameters as well as the 95% high density interval (HDI) which reports the range of 95% of the posterior distribution [45]. Due to forced-choice in the questionnaires there was no missing data. For correlation analysis with gender, the participant in the category “other” was eliminated from the dataset.

## Results

### Qualitative results

#### The categories

In our qualitative content analysis we found three main categories of barriers: social, formality, and job demand problems. Social problems include any obstacle concerning communication and human interaction. Formality problems sum up problems with organizational and practical process-related aspects of the job entry. Job demand problems describe difficulties with meeting specific requirements of an employment.

Regarding solutions, we found two main categories: self-solution and external help. Self-solutions sum up different coping strategies of the individual. Solutions with external help include all approaches where the individual sought support.

Tables 2 and 3 give an overview of the complete category system including definitions of categories and corresponding examples.

**Table 2:** Category system for responses concerning expected and occurred barriers.

General category	Sub-category	Definition	Response example
Social problems	colleagues	Interaction with colleagues	„working in a team“
	communication	General communication; non-personal communication in application process	„misunderstandings in social communication“
	customers	Interaction with customers	„clients complained about too little contact”
	handling the diagnosis	Problems regarding autism-typical behavior and its handling	„prejudices against severe disabilities“
	interview	Communication problems in job interviews	„job interviews (unsecure manner, wrong responses to questions)“
	mobbing	Mobbing, verbal and physical attacks	„animosities, mobbing, physical violence“
	supervisors	Interaction with supervisors	„missing / insufficient personal contact with […] supervisors“
	other	Other social situations	„christmas parties, birthdays, etc.“
Formality problems	agencies	External organizations: authorities, non-profit associations, civil service	„no help from the job center“
	application process	Finding matching job vacancies; creating applications	„mean effort of 7h for one cover letter“
	equipment and environment	Work setting and sensory influences with concrete cause	„placement into an open plan office“
	work routine	Plans and working structures defined externally; hierarchy	„unclear work instructions“
	qualification	Professional suitability; CV	„rejection because of missing job experience“
	support	Orientation period and contact person	„not enough guidance”
	other	Other formal requirements	„age, gender“
Job demand problems	cognitive	Skills; capabilities	„I cannot or barely multitask“
	stress & psychosomatic	Stress and its emotional and physical consequences	„loneliness, dejectedness, headache, backache“
	time-related	Mismatch between personal rhythm and work rhythm	„feeling of no leisure time left because of commuting“
	other	Other job demands	„moving into a new city“

**Table 3:** Category system for responses concerning solutions.

General category	Sub-category	Definition	Response example
Self-solutions	acceptance	Showing strength in perseverance	„with a lot of patience“
	avoidance / resignation	Escaping from / giving up on a situation and suffering from it	„I suffered and held my tongue“
	communication	Approaching colleagues or superiors proactively	„Asking further questions until everything is clear“
	compensation	Using strengths to compensate	„strengths in other areas“
	concealment of diagnosis	Hiding diagnosis; lying about it	„lying with general requirements (flexibility, resilience etc.)“
	information about diagnosis	Making the diagnosis an open issue	„I told my supervisor of my diagnosis. He took it well.“
	practice / qualification	Intellectual solution in form of trouble-shooting and seeking more information	„application training“
	other	Self-solution not otherwise specifiable; compromise; independency; luck	„self-employed, with home office“
External help	external institutions	External organizations: authorities, non-profit associations, civil service	„usage of integrational service“
	private environment	Family, friends, acquaintances	„my parents helped me“
	work environment	Colleagues, superiors	„reduction of working time“
	other	Help from others, not otherwise specified	„I had support“

#### Expected barriers

Participants gave a total of 242 answers to the question which barriers they expected before entering the job market. For individuals in non-autism-specific employment the most frequent problem fell into the category of social problems of communication (15%), followed by the formality problems of equipment and environment (12%), work routines (10%), application process (10%), and qualification (8%) (see Table 4). Participants with autism-specific employment pointed out the formality problem of qualification as the most frequent problem (23%), then the social problem of communication (11%), followed by the formality problems of equipment and environment (9%), work routine (9%), and cognitive job demand problems (9%) (see Table 5). The ratio of the general categories in both groups can be seen in Table 6. Individuals with autism-specific employment expected more formality problems and more job demand problems, but less social problems than individuals with no such specific employment. Job demand problems were the least frequent in individuals in non-autism-specific employment, but not in individuals with autism-specific employment.

**Table 4:** Absolute and relative response frequency for participants without autism-specific job.

Question type	General category	Sub-category	N	%	N	%
Barriers			Expected barriers		Occurred barriers	
	Social problems	colleagues	14	7	19	7
		communication	28	15	43	15
		customers	8	4	10	3
		handling the diagnosis	9	5	14	5
		interview	12	6	14	5
		mobbing	2	1	11	4
		supervisors	3	2	8	3
		other	3	2	2	1
	Formality problems	agencies	5	3	6	2
		application process	18	10	21	7
		equipment and environment	23	12	47	16
		qualification	15	8	6	2
		support	5	3	10	3
		work routine	19	10	38	13
		other	2	1	4	1
	Job demand problems	cognitive	10	5	15	5
		stress & psychosomatic	8	4	19	7
		time-related	1	1	1	0
		other	4	2	2	1
			Used solutions			
Solutions	Self-solution	acceptance	27	13		
		avoidance / resignation	27	13		
		communication	18	9		
		compensation	4	2		
		concealment of diagnosis	4	2		
		information about diagnosis	9	4		
		practice / qualification	19	9		
		other	27	13		
	External help	external institutions	14	7		
		private environment	3	1		
		work environment	46	22		
		other	8	4		

**Table 5:** Absolute and relative response frequency for participants with autism-specific job.

	No autism-specific job	Autism-specific job
Expected problems		
Social problems	35%	22%
Formality problems	44%	50%
Job demand problems	21%	27%
Occurred problems		
Social problems	35%	15%
Formality problems	44%	66%
Job demand problems	21%	19%
Solutions		
Self-solution	51%	52%
External help	49%	48%

**Table 6:** General categories for expected problems, occurred problems and solutions in individuals with no autism-specific job vs. with autism-specific job.

Question type	General category	Sub-category	N	%	N	%
Barriers			Expected barriers		Occurred barriers	
	Social problems	colleagues	1	2	6	9
		communication	6	11	4	6
		customers	1	2	0	0
		handling the diagnosis	3	6	2	3
		interview	1	2	0	0
		mobbing	0	0	0	0
		supervisors	2	4	0	0
		others	1	2	0	0
	Formality problems	agencies	2	4	1	1
		application process	4	8	11	16
		equipment and environment	5	9	12	18
		qualification	12	23	7	10
		support	1	2	7	10
		work routine	5	9	8	12
		others	0	0	1	1
	Job demand problems	cognitive	5	9	4	6
		stress & psychosomatic	3	6	2	3
		time-related	0	0	1	1
		others	1	2	1	1
			Used solutions			
Solutions	Self-solution	acceptance	12	21		
		avoidance / resignation	0	0		
		communication	13	23		
		compensation	2	4		
		concealment of diagnosis	0	0		
		information about diagnosis	1	2		
		practice / qualification	7	12		
		other	2	4		
	External help	external institutions	2	4		
		private environment	4	7		
		work environment	10	18		
		other	4	7		

*Note*. Irrelevant answers were not included in this analysis. All relevant general categories were relativised by the number of sub-categories they contained, making the coding of each general category equally probable.

#### Occurred barriers

Participants named 357 barriers they encountered. The formality problem of equipment and environment was the most common one for individuals without (16%, see Table 4) and with autism-specific employment (18%, see Table 5). However, while this was followed by the social problem of communication (15%) and the formality problem of work routine (13%) in non-autism-specific employment, the second and third most frequent problems for individuals in autism-specific employment were also formality problems, namely application processes (16%), and work routines (12%).

For individuals in non-autism-specific employment the expectations and the occurrence of problems showed the same ratio of general categories (see Table 6). Individuals with autism-specific employment, however, faced more formality problems and less job demands and social problems than expected. They also faced fewer problems than they expected (59%) compared to individuals in non-autism-specific employment (72%, see Table 7). In a Bayesian inference test 95.8% of the posterior distribution are in favor of the difference.

**Table 7:** Absolute and relative frequency of cross-question cases for participants without vs. with autism-specific job.

	NASE		ASE	
	N	%	N	%
Occurrence of expected problems				
The expected problems occurred.	106	72	27	59
The expected problems did not occur.	41	28	19	41
Solution of occurred problems				
The occurred problems were solved.	129	55	33	61
The occurred problems were not solved.	105	45	21	39

*Note*. NASE = individual with non-autism-specific employment; ASE = individual with autism-specific employment. Only responses without missing data were included in this analysis.

#### Solutions

In total, 263 solutions were named. The most frequent solution in individuals in non-autism-specific employment was external help from the work environment (22%, see Table 4). Individuals working in autism-specific employment however named the self-solutions communication (23%) and acceptance (21%) as their most frequent approach to solving problems, even before using external help from the work environment (18%, see Table 5). The overall proportion between self-solutions and external help was similar in both groups (see Table 6). Yet in sum, individuals in autism-specific employment solved a slightly higher proportion of occurred problems (61%) than individuals in non-autism-specific employment (55%, see Table 7). In a Bayesian inference test, 78.1% of the posterior distribution are in favor of the difference.

#### Rating

Participants in non-autism-specific jobs rated the social problems as most important to them (43%), followed by formality problems (30%), and job demand problems (27%). These general categories had the same order in the second priority participants rated (50%, 37% and 13%, respectively). Yet in the third priority, formality problems were named most frequently (70%), followed by job demand problems (20%), and then social problems (10%).

Participants in autism-specific employment, however, put higher emphasis on formality problems (60%) than on social problems (40%) in the first priority. In the second priority, this ratio changes to 48% / 52%. Job demand problems are only mentioned as a third priority and are the most frequently named (48%) before formality problems (28%) and social problems (24%).

### Quantitative results

Results for mean values and standard deviance as well as all bivariate correlations can be found in Table 8. There was a correlation between gender and occupational self-efficacy (*r* = -.29, see Table 8) with males tending to have higher occupational self-efficacy than females. The same tendency was seen for gender and general self-efficacy (*r* = -.20).

**Table 8:** Summary of the bivariate Bayesian correlations of all variables.

	male	female	1	2	3	4	5	6	7	8	9	10
(1) Gender	N = 29	N = 36										
	NASE	ASE										
(2) Employment group	N = 49	N = 17	-^b^	1								
	**M**	**SD**										
(3) Tenure	4.76	6.52	.07	-.25	1							
			[-.22;.35]	[-.49;25]								
(4) GSE	2.22	0.58	-.20	.28	-.06	1						
			[-.42;.05]	[.01;.52]	[-.31;.20]							
(5) OSE	2.32	0.67	-.29	.24	.01	.83	1					
			[-.51;.05]	[.01;.47]	[-.24;27]	[.73;.89]						
(6) LS	2.65	1.07	-.09	.07	.17	.44	.45	1				
			[-.35;.14]	[-.17;.32]	[-.10;.42]	[.22;.62]	[.24;.64]					
(7) JS^a^	0.00	0.88	.02	.00	.10	.30	.38	.65	1			
			[-.23;.26]	[-.25;.24]	[-.17;.36]	[.06;.52]	[.16;.58]	[.49;.78]				
(8) improvement	3.41	1.35	-.09	.08	.01	.33	.32	.38	.61	1		
			[-.34;.16]	[-.17;.32]	[-.26;27]	[.09;.54]	[.10;.53]	[.16;.58]	[.44;.76]			
(9) job importance	4.11	1.23	-.17	.18	-.01	.41	.42	.35	.23	.34	1	
			[-.41;.08]	[-.06;43]	[-.28;26]	[.18;.61]	[.20;.62]	[.12;.55]	[-.02;.45]	[.09;.56]		
(10) strengths	3.39	1.40	-.13	.16	.09	.45	.48	.62	.81	.60	.29	1
			[-.37;.11]	[-.08;.39]	[-.17;.35]	[.24;64]	[.27;.66]	[.45;.76]	[.72;.90]	[.42;.75]	[.05;.51]	

*Note*. 95% high-density intervals are displayed in brackets; NASE = non-autism-specific employment; ASE = autism-specific employment; GSE = general self-efficacy, OSE = occupational self-efficacy, LS = life satisfaction, JS = job satisfaction, improvement = control item regarding quality of life improvement durinc current employment, job importance = control item regarding the importance of a job for the quality of life, strengths = control item regarding the use of strengths in current employment,

^a^ = standardized z-score,

^b^ = left out of data-analysis due to one employment group being an all-male-group.

General self-efficacy was correlated with the employment group (*r* = .28), general self-efficacy being higher with individuals in autism-specific employment. There was a correlation between occupational self-efficacy and the employment group (*r* = .24) insofar as individuals with autism-specific employment tended to have higher occupational self-efficacy.

Correlations of all control items with general self-efficacy, occupational self-efficacy, life satisfaction, job satisfaction, and the other control items were moderate to strong. The strengths item, asking whether personal strengths are used in current employment, showed the highest correlations, namely with general self-efficacy (*r* = .45), occupational self-efficacy (*r* = .48), life satisfaction (*r* = .62), and job satisfaction (*r* = .81).

## Discussion

The main purpose of this study was to discover successful ways of entering the job market for individuals with autism by identifying barriers they may have faced and solutions they may have used to overcome them. We thereby also sought to examine positive work-related variables that could be of importance in the employment process.

In the first step of our study we identified a wide range of barriers that individuals with autism expected and encountered. Some of them, like filling out job applications, job search, communication and interaction with supervisors had also been reported by Müller, Schuler [10]. By contrast, we created a system of perceived barriers that is both more general and more specific through its structure of general categories and sub-categories.

The higher frequency of social problems in non-autism-specific employment and of formality problems in autism-specific employment suggests a difference in the nature of the barriers individuals with autism encounter when entering the job market. The two groups did not just differ in the number of certain problems that occurred but also in how they rated their importance. Individuals with non-autism-specific employment rated social problems as more important than formality problems while the contrary occurred with individuals with autism-specific employment. In that regard, the most frequent problems were also seen as the most important ones. However, even though individuals in autism-specific employment faced more job demand than social problems, they rated job demand problems as less important. This is relevant because it shows that in practice, social problems should not be neglected when they are less frequent.

Our findings suggest that individuals in different types of employment face qualitatively different barriers. Hagner and Cooney [51] found that supervisors reported direct communication as an important strategy for successful employment of individuals with autism. The use of this strategy might have led to the small number of communication problems for individuals in autism-specific employment. At the same time, their skill set not matching their job content (formality problem–qualification) might hinder successful long-term employment [52]. Problems concerning work routines occurred in both groups and could be solved by introducing more structure in schedule and responsibilities [51]. Many participants of both groups also named the equipment and work environment as problems, for instance criticizing the noise level in open-plan offices. Since many individuals with autism have shown high sensitivity to sensory input like noise and light [53–56] and participants in our study frequently reported such problems, reducing distracting stimuli through the creation of individual workspaces seems important and necessary. All of these findings present some form of adaptation to the needs of individuals with autism. Hence we endorse the idea of Mawhood and Howlin [52] that a successful approach towards employment of individuals with autism is based on an appropriate work setting and understanding of their individual needs.

Regarding solutions, we found different patterns in the two study groups that might provide further ideas for this approach. Individuals in autism-specific employment tended to solve occurring problems less with resignation and more with acceptance, communication, and practice or further qualification. This is interesting because impairment in communication is a core symptom of Autism Spectrum Condition [4] and has been named as a cause of difficulties in the employment process [10, 57]. Yet for participants in autism-specific employment this reported weakness was not just attenuated but even transformed into a resource of problem solving behavior. Maybe this was facilitated by being surrounded by peers or a supervisor’s adjustment towards more direct communication [51].

Communication, as well as the self-solutions acceptance and practice/qualification, matches the description of active coping given by Carver, Scheier [58], as opposed to avoidance coping (methods of resignation and denial). Avoidance coping has shown to be less effective than active coping [59] and correlates with psychological strains [60]. Active coping, however, has shown relations with optimism [61], hope [62, 63], and resilience [64–67]. Thus these constructs, too, seem to be important strengths for active problem solving. It is for further studies to examine how individuals with autism could identify their strengths, how they might relate to their coping behavior and how they might even pave the way for more long-term and prevention-oriented solutions [68].

The second part of our study focused on positive correlates of employment and the type of employment in individuals with autism. Concerning the relation of employment groups with general and occupational self-efficacy, our results showed small correlations and medium effects between autism-specific employment and general and occupational self-efficacy.

These differences in self-efficacies between individuals with and without autism-specific employment could have been found for several reasons. A possible explanation might be that self-efficacy is an effect resulting from employment in an autism-specific company. Its system of on-the-job support might create a protected environment in which employees are fostered and experience mastery. It has been suggested that self-efficacy is influenced by past experiences of mastering a situation and external appraisal [17, 18, 23, 69, 70]. Thus individuals in autism-specific employment might have had more of these experiences and were more positively appraised by supervisors or job coaches, resulting in a higher general and occupational self-efficacy. It might be that it is related to mastering the demanding entry process in the autism-specific company.

Furthermore, one could hypothesize that job demands in the autism-specific company, located in the IT sector, were particularly high, thus attracting only individuals already seeing themselves as very self-efficient. At the same time, the autism-specific company aims at employing individuals with autism based on their strengths while being a regular competitive business. When their selection procedures are highly demanding and based on testing for relevant strengths, it is possible that the selected employees are those with highest cognitive capacities and also highest self-efficacies.

Our finding of a correlation between occupational self-efficacy and gender could serve as another explanation for the group differences, since only males were in autism-specific employment. This distribution over the groups might be due to a generally higher interest in STEM subjects and jobs in males reported for neurotypical adults [71, 72]. No numbers on gender disparity in occupational fields for individuals with autism have yet been assessed.

However, the implications of the gender distribution for our findings have to be discussed. There have been reports of higher general self-efficacy in neurotypical males compared to females, but these effects were small or statistically not significant [73–75]. Considering the fact that we found no statistically significant correlation between general self-efficacy and gender, we conclude that gender does not account for the differences between individuals with and without autism-specific employment regarding general self-efficacy.

For the higher occupational self-efficacy in individuals in autism-specific employment we argue that the occupational field of this group as well as the gender might be responsible because neurotypical males show higher task-specific self-efficacy in tasks involving IT and computers [76–79]. Further assessment could evaluate whether the nature of the task’s relation to occupational self-efficacy is influenced by gender.

In our comparison of qualitative and quantitative results we saw that self-efficacy could also be linked to coping behavior. Jex, Bliese [60] found the correlation of self-efficacy to be negative with avoidance coping and positive with active coping, which is consistent with our results. It would be important to examine the direction of this possible effect in order to see whether self-efficacy is a strength that encourages active coping or the result of effective coping experiences. In conclusion, we can only speculate for possible causal connections from our correlations. Only experimental or longitudinal studies can test the underlying mechanisms.

However, even though our results indicated that participants in autism-specific employment showed higher general self-efficacy than those in non-autism-specific employment, it is still important to note that general self-efficacy is more than one standard deviation below the mean of neurotypical adults [M = 29.59, SD = 5.29, N = 18,000, based on the dataset of [80] compared to M = 22.17, SD = 5.82 in our total sample]. This is also consistent with the findings of Lorenz and Heinitz [81], who found differences of more than one standard deviation for general and occupational self-efficacy between individuals with autism and neurotypical individuals.

Factors influencing self-efficacy may be within individuals’ control or outside of it [17], although resources such as knowledge or skills can be used when available or can otherwise be acquired. One could suggest that individuals with autism have lower self-efficacy because they have fewer resources. One could also suggest that acquisition of resources can prove more difficult for them than for neurotypical individuals, due to a lack of embodied empathy [82], possibly indicating impaired vicarious learning. Therefore, some resources may be less controllable for individuals with autism.

As stated earlier, mastery of past experiences and external appraisal are potential antecedents of self-efficacy [17, 18, 23, 69, 70]. We argue that individuals with autism face many difficulties in education and work life [10, 83] and lack positive experiences of mastery because these problems are rarely solved by supporting parties (see[84] for an overview). Moreover, stigma and subsequent focus on weaknesses as well as a lack of external appraisal of individuals with autism might also lower their self-efficacy, as it does in other groups facing prejudices [85, 86].

General self-efficacy’s positive relation with job performance [20, 87], health [21], and subjective well-being [22] was mentioned earlier. Its connection to life satisfaction, job satisfaction, and improvement of quality of life (improvement item) has been indicated in our study. Self-efficacy is also a factor introduced by Luthans, Luthans [69] as one of four components of the positive psychological capital, a construct recently suggested to be crucial in employee well-being and satisfaction [88]. It therefore seems of high importance to thoroughly investigate the discrepancy in self-efficacy between individuals with and without autism. Furthermore, it may even extend the search for possible unused strengths to the other components of positive psychological capital, namely optimism, hope and resilience [69].

While our results showed moderate to strong correlations of life and job satisfaction with general and occupational self-efficacy, respectively, employment groups only differed in both self-efficacies, but not in satisfaction. We argue that this is due to the high amount of problems in both groups, leading to a similar level of satisfaction. At the same time, even though the employment groups reported different types of occurred problems (i.e. more social or formality problems), their life and job satisfaction did not differ. Thus, quality of barriers may not influence satisfaction measures. What might influence satisfaction measures is one’s personal evaluation of whether a job is important for one’s quality of life (job importance item). We found that participants who evaluated their job as important for their quality of life had higher life satisfaction but not job satisfaction. Emphasizing a job when evaluating quality of life may sensitize individuals with autism and trigger demands towards their job. Further research is needed to address possible implications of this finding on the employment of individuals with autism.

In sum, the two groups of individuals with and without autism-specific employment showed differences in quality of occurred barriers, quality of coping strategies, levels of general and occupational self-efficacy, but not in life or job satisfaction. Based on these findings we proposed a more customized approach to successfully employ individuals with autism. Employment should be based on their needs and their resources. Our results should encourage individuals with autism, employers and support workers alike to focus on strengths and solutions instead of deficits. While we find it important to address specific problems and barriers that occur, we think that strengths should be identified and fostered at the same time. This is crucial to our approach of positive organizational psychology and could not only facilitate concrete problem solving, but also enhance self-efficacy.

However, before designing practical applications, the next aim must be to investigate barriers and solutions quantitatively in order to assess their relations to coping strategies, self-efficacies as well as life and job satisfaction. We are positive that the present study is one step towards a better understanding of possible employment and well-being for individuals with autism.

## Limitations

The results of this study should be interpreted with the following limitations in mind. First, the participants were recruited and participated online. Therefore, the study may have only reached certain individuals and lack generalizability. According to Gosling, Vazire [89], however, the online recruitment should only be of marginal effect to the results. Also, all participants were employed and thus can only indicate a reflection of the individuals with autism that successfully applied for a job. Individuals who did not succeed were not part of the study. More concerns about generalizability are warranted because this study used a nonprobability sample. Furthermore, participants were all of German-speaking descent, and were therefore relatively ethnically homogeneous.

The male/female ratio in the study was 0.8:1. This is contrary to the male/female ratio in the overall population of individuals with autism, currently estimated as 4.4:1 [5]. A possible explanation may lie in the reported sex differences regarding symptom severity: women with autism show less repetitive and stereotyped behavior as well as less impairment in externalization and executive functions [90–93]. Thus, they might master social situations in the employment process more easily and therefore have higher chances of employment.

The participants in the non-autism-specific employment sample were not diagnosed by means of a singular diagnostic method. Instead, they were asked to provide information about their autism diagnosis. Because of the strong variation within the diagnostic process, we had to rely upon the participants' self-reported data of an existing diagnosis.

### Ethics Statement

This study is in accordance with the APA ethical principles regarding research with human participants. This study does not involve any conflict of ethics, since no clinical intervention was performed. Neither were blood or tissue samples taken for study purposes.

Participants were informed before participating that their responses would be treated confidentially and anonymously and that all data would be analyzed in a generalized manner so that no conclusions could be drawn about individual persons. The participants were informed that they would give their consent by proceeding past the welcome page of the online survey. This procedure is in accordance with the Freie Universität Berlin ethics committee’s guidelines. There was no contact between researchers and participants. Participation in this study was completely voluntary. This study was approved by the ethics committee of Freie Universität Berlin ID 101/2015.

## Supporting Information

S1 Appendix Interview questionnaire on barriers to employment and their overcoming. (DOCX)

S1 Dataset Complete dataset of all variables used in quantitative analysis. (XLSX)
