# Supplementary material for: Autism and Overcoming Job Barriers: Comparing Job-Related Barriers and Possible Solutions in and outside of Autism-Specific Employment
Source: PLoS One. 2016 Jan 14;11(1):e0147040. doi: 10.1371/journal.pone.0147040 (PMC4713226; doi:10.1371/journal.pone.0147040)
Supplement: S1 Appendix — (DOCX) [file pone.0147040.s001.docx]

**S1 Appendix**

**Questionnaire on barriers to employment and their overcoming**

1. Did you expect to have any problems with the general process of job-seeking?
   1. No.
   2. Yes, namely:
2. What problems occurred during the general process of job-seeking?
   1. None.
   2. The following:
3. If applicable, how did you resolve these problems?
4. Did you expect to have any problems when drafting your application?
   1. No.
   2. Yes, namely:
5. What problems occurred during the creation of your application?
   1. None.
   2. The following:
6. If applicable, how did you solve these problems?
   1. ­
7. Did you expect to have any problems with regard to the contact with potential employers?
   1. No.
   2. Yes, namely:
8. What problems occurred regarding the contact with potential employers?
   1. None.
   2. The following:
9. If applicable, how did you solve these problems?
   1. ­
10. Did you expect to have any problems with regard to the demands of each job?
    1. No.
    2. Yes, namely:
11. What problems occurred regarding the job demands?
    1. None.
    2. The following:
12. If applicable, how did you solve these problems?
    1. ­
13. Did you expect to have any problems with regard to the working day?
    1. No.
    2. Yes, namely:
14. What problems occurred regarding the work routine?
    1. None.
    2. The following:
15. If applicable, how did you solve these problems?
    1. ­
16. Did you expect to have any problems with regard to the workplace equipment?
    1. No.
    2. Yes, namely:
17. What problems occurred regarding the equipment of your workplace?
    1. None.
    2. The following:
18. If applicable, how did you solve these problems?
    1. ­
19. Did you expect to have any problems within the work environment?
    1. No.
    2. Yes, namely:
20. What problems occurred regarding the work environment?
    1. None.
    2. The following:
21. If applicable, how did you solve these problems?
    1. ­
22. Did you expect to have any problems with the support mechanisms at the workplace?
    1. No.
    2. Yes, namely:
23. What problems occurred regarding the support at the workplace?
    1. None.
    2. The following:
24. If applicable, how did you solve these problems?
    1. ­
25. Did you expect to have any problems other than those mentioned previously
    1. No.
    2. Yes, namely:
26. What problems that are not mentioned so far occurred?
    1. None.
    2. The following:
27. If applicable, how did you solve these problems?
    1. ­
28. Please name one to three possible problems, that you consider the most challenging. Name the most challenging first. This question helps us identify the potential need for certain measures.
